# P12 Clinical perceptions of the need for anaerobic coverage in patients treated for aspiration pneumonia (AP)

**DOI:** 10.1093/jacamr/dlae136.016

**Published:** 2024-08-23

**Authors:** Simon Dewar, Natalie Lee, Uladzimir Antonenka, Tamanna Botsani, Maya Brown, Omar Khoja, Angel Sin Yu Lam, Harry Lawson, Chirag Shetty, Natasha Vijendren, Lok Yiu (Charlotte) Yung, Lucia Zilles

**Affiliations:** NHS Lothian, UK; NHS Lothian, UK; University of Edinburgh Medical School, Edinburgh, UK; University of Edinburgh Medical School, Edinburgh, UK; University of Edinburgh Medical School, Edinburgh, UK; University of Edinburgh Medical School, Edinburgh, UK; University of Edinburgh Medical School, Edinburgh, UK; University of Edinburgh Medical School, Edinburgh, UK; University of Edinburgh Medical School, Edinburgh, UK; University of Edinburgh Medical School, Edinburgh, UK; University of Edinburgh Medical School, Edinburgh, UK; University of Edinburgh Medical School, Edinburgh, UK

## Abstract

**Background:**

In 2023, the British Thoracic Society (BTS) published a clinical statement on management of patients with aspiration pneumonia (AP). As part of the recommendations, it was highlighted that routine anti-anaerobic coverage is not required for patients treated for AP, except in specific clinical circumstances. However, hospital inpatients treated for AP are frequently antimicrobial regimens that include metronidazole.

**Objectives:**

This small study aimed to garner the perceptions of prescribing clinicians regarding antimicrobial prescribing for AP.

**Methods:**

A questionnaire was devised, consisting of seven questions based on a Likert scale (1=strongly agree, 5=strongly disagree), and the option to provide additional qualitative comments. This was distributed to healthcare professionals at the Western General Hospital, Edinburgh in February 2024. Specialties targeted were Medicine of the Elderly, General Medicine and Respiratory Medicine. Eighteen responses were returned, with 50% of responders being Foundation grade doctors.

**Results:**

Monotherapies for AP were generally disfavoured by respondents, although there was more confidence in using co-trimoxazole monotherapy over either amoxicillin or doxycycline. However, there was no clear consensus about the need for metronidazole as part of treatment regimens. This was supported by the qualitative statements made by respondents. Only 38% of respondents felt that AP did not require initiation of antimicrobials in all circumstances. Additional comments from respondents also offered insights into specialty-specific decision-making. There was a particular preference amongst doctors working in Medicine of the Elderly for using metronidazole co-therapy in treatment of AP, in part due to perceived severity of infection in their patient population. 87% of respondents reported that they use NHS Lothian’s antimicrobial companion app to support their prescribing decisions. This app has the potential to be utilized as an educational tool for junior doctors with regards to antimicrobial prescribing.

**Conclusions:**

This small study highlights that there is still the perception that metronidazole is required in treatment of AP, and that monotherapy is generally disfavoured by prescribing clinicians. Further work in this area could include targeted quality improvement projects and education in the areas most likely to treat patients with AP.

